# Impact of histone modifier-induced protection against autoimmune encephalomyelitis on multiple sclerosis treatment

**DOI:** 10.3389/fneur.2022.980758

**Published:** 2022-10-14

**Authors:** Sundararajan Jayaraman, Arathi Jayaraman

**Affiliations:** ^1^Department of Surgery, University of Illinois College of Medicine, Peoria, IL, United States; ^2^Xavier University School of Medicine, Oranjestad, Aruba

**Keywords:** central nervous system, epigenetics, experimental autoimmune encephalomyelitis, histone deacetylases, multiple sclerosis, myelin oligodendrocyte glycoprotein, neutrophils, T helper cells, tolerance

## Abstract

Multiple sclerosis is a progressive demyelinating central nervous system disorder with unknown etiology. The condition has heterogeneous presentations, including relapsing-remitting multiple sclerosis and secondary and primary progressive multiple sclerosis. The genetic and epigenetic mechanisms underlying these various forms of multiple sclerosis remain elusive. Many disease-modifying therapies approved for multiple sclerosis are broad-spectrum immunomodulatory drugs that reduce relapses but do not halt the disease progression or neuroaxonal damage. Some are also associated with many severe side effects, including fatalities. Improvements in disease-modifying treatments especially for primary progressive multiple sclerosis remain an unmet need. Several experimental animal models are available to decipher the mechanisms involved in multiple sclerosis. These models help us decipher the advantages and limitations of novel disease-modifying therapies for multiple sclerosis.

## Introduction

### Clinical manifestations of multiple sclerosis

More than 2.8 million people live with multiple sclerosis (MS) worldwide, and the prevalence has been increasing (1). The mean age of diagnosis of MS is 32 years, with twice the number of female patients compared with male patients afflicted with this disease. However, the basis of sexual dimorphism in MS manifestation remains elusive, as in other autoimmune diseases. MS is a prototypical organ-specific autoimmune disease of the central nervous system (CNS), affecting the brain and spinal cord (2–4). Most (85%) patients with MS manifest relapsing-remitting MS (RRMS), characterized by alternate periods of relapses and remissions for decades after an initial episode of neurological dysfunction, clinically isolated syndrome. Relapses accompany CNS inflammation and demyelination detectable as white matter lesions by magnetic resonance imaging. Accumulating disabilities during relapses in most (80%) patients with MS leads to secondary progressive MS (SPMS), characterized by decreased brain volume and increased axonal loss without associated inflammatory lesions. A minor fraction (10%) of patients with MS continue to decline progressively from the beginning of diagnosis without relapses. Variations of MS include progressive-relapsing and pediatric disease and severe Marburg variant. The hallmark of MS is sharply demarcated demyelinating plaque with axons relatively preserved, whereas in neuromyelitis optica (MNO), both axons and myelin are involved, resulting in necrotic cavitation. Severe involvement of optic nerves and the spinal cord is a characteristic of the opticospinal MS (OSMS) subtype, which is more prevalent in African Americans (5, 6). Compared with Whites, African Americans had an older age at onset, experienced greater disability, progressed faster, had increased risk for SPMS, experienced transverse myelitis more often, and were likely to have motor symptoms and the OSMS subtype. The classic multifocal MS is rare in Japanese, who manifest OSMS with features similar to those of the relapsing form of NMO in Western populations, and was proposed to be the same as the NMO disorder, rather than a form of MS (7). However, in Brazilian patients, OSMS is recognized as a milder MS phenotype distinct from NMO (8). While antibody-dependent aquaporin four loss occurred in some patients with NMO, antibody-independent astrocytopathy was found in several demyelinating conditions, including Baló's disease, NMO, and MS (9). In addition to these complexities, MS is also rare among Samis, Turkmen, Uzbeks, Kazakhs, Kyrgyzis, native Siberians, North and South Amerindians, Chinese, Japanese, African blacks, and New Zealand Maoris, in contrast to a high propensity of Sardinians, Parsis, and Palestinians to develop MS (10). The different susceptibilities of distinct racial and ethnic groups are essential determinants of the uneven geographic distribution of MS.

**Figure 1:**
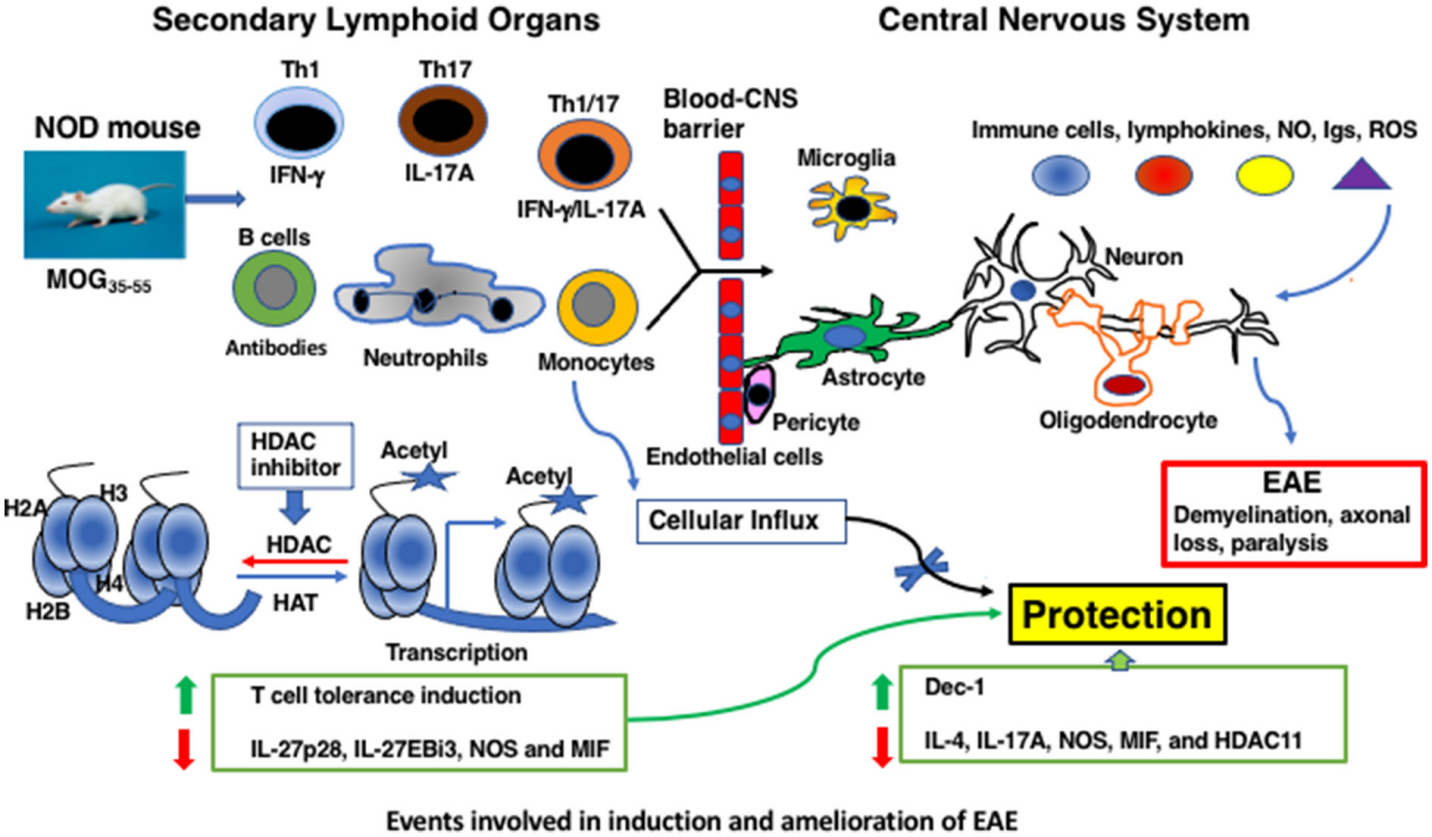
Events involved in induction and amelioration of EAE.

The clinical manifestations of MS include temporary vision loss, sensory and motor problems, fatigue, impaired bowel and sexual functions, cognitive deficits, and paralysis (2–4). Distinct forms of MS appear to correlate with the spatiotemporal dissemination of lesional sites within the CNS (2–4, 11). The hallmarks of MS pathology include the breakdown of the blood–brain barrier, accumulation of immune cell infiltrates, oligodendrocyte loss, demyelination, astrogliosis, axonal degeneration, and disruption of neuronal signaling (Figure 1). Substantial T-cell infiltration occurs in patients with acute and relapsing disease but is spared during later stages of MS, despite an unabated neuronal disability. Intrinsic neuronal deficits such as those associated with Alzheimer's disease are thought to play a role, especially during the advanced stage of MS (11).

### Genetics of MS

Although the etiology of MS remains elusive, genes within the human leukocyte antigenic (HLA) loci, such as HLA-A^*^02:01, HLA-DRB1^*^15:01, HLA-DRB5, HLA-C, and *TNF*, have been firmly associated with MS susceptibility (12). In African Americans, classic/multifocal MS is associated with DRB1^*^15 alleles, whereas OSMS is not (5). Not only the DRB^*^1501 allele but also the extended DRB1^*^1501-DQB1^*^0602 haplotype is commonly found in northern Europeans with MS (5). The HLA-DPB1^*^0501 haplotype is not uniquely associated with the OSMS subtype, which is relatively more common in Japan (13). Interleukin-2 (IL-2) and its receptor IL-2R play a crucial role in MS and are also crucial for T-cell tolerance (14). In addition, the soluble form of the IL-2R (sIL-2R) plays a role in MS. IL-7 and IL-7Rα form a non-redundant ligand–receptor system and plays a critical role in T-cell activation. Peripheral blood mononuclear cells of patients with MS display deletion of exon 4 of the IL-7 transcript and splice variants lacking exons 5, 6, and 7 (15). A closer analysis of the impact of these genetic variations is necessary for a better understanding of MS pathogenesis.

### The pivotal role of T helper cells in MS

Cerebrospinal fluid (CSF)-infiltrating CD4^+^ T cells of patients with MS proliferated and secreted interferon- γ (IFN- γ), a characteristic of the Th1 subset, but not IL-17 when challenged with the myelin oligodendrocyte glycoprotein 35–55 (MOG_35 − 55_) peptide *in vitro* (16), a proposed candidate CNS determinant in MS (17). However, others reported the abundance of IL-17-expressing Th17 cells in the peripheral blood, CSF, and brain lesions of patients with MS, which increased during relapses (18). Increasing evidence also indicates a role of central memory Th17.1 (Th1/17) cells, which share the hallmarks of Th1 and Th17 cells, respectively, in IFN- γ and IL-17 production, in MS (18). In addition to Th17 cells, follicular helper T cells that promote the germinal center formation, B-cell differentiation, and antibody production are also implicated in several autoimmune diseases, including MS (19). The intrathecal inflammatory environment in patients with RRMS promotes the recruitment of peripheral follicular helper T cells to the CNS without increasing their ability to migrate (20). Since the follicular helper T cells failed to transfer demyelinating disease in mice (21), it is unlikely that they have pathological consequences in patients with MS. The role of follicular T helper cells in MS remains to be proven. Although MHC class I-restricted CD8^+^ cells were found in the brain lesions of patients with MS, they were also present in patients with infections and other brain diseases, providing inconclusive evidence for their involvement in MS (22).

### Epstein–Barr virus (EBV) infection and MS

Infection with EBV is associated with monoclonal or oligoclonal B-cell expansion in many autoimmune diseases, including Hashimoto's thyroiditis, Grave's disease, Sjögren's syndrome, rheumatoid arthritis, systemic lupus erythematosus syndrome, and MS (23). Whereas infectious mononucleosis increases the risk of MS, the vast majority (90–95%) of the world population infected with EBV at some point in life does not develop MS (24). Although elevated EBV nuclear antigen 1 IgG titers are associated with gadolinium-enhancing brain lesions, the lack of correlation between acute viral reactivation in the peripheral blood and MS lesions suggests a limited role for EBV infection in driving the disease activity (25). Despite the increased level of EBV viral load in patients with RRMS compared with controls, there was no statistically significant difference in EBV and human herpes virus-6 (HHV-6) copy numbers between the patients and controls (26). In addition, the frequency of NK and CD8^+^ T cells increased during relapse, which was not associated with EBV and HHV6 plasma viral loads. Although EBV infection has been hypothesized to contribute to MS development in the context of other predisposing conditions, such as the HLA genotype, vitamin D deficiency, smoking, and altered T-cell responses (23), evidence for this hypothesis remains to be garnered.

### Disease-modifying therapies for MS

Several disease-modifying therapies (DMTs) were approved for MS treatment by the Food and Drug Administration (FDA) [reviewed in (27, 28)]. These include self-injectables such as the anti-virals IFN-β-1a and b, first-line treatment, and peginterferon beta-1a provided moderate protection against RRMS (29, 30). However, the production of antibodies against IFN-β and the lack of the effect on Th17 cells, considered encephalitogenic, (31) remained a major concern. Glatiramer acetate designed based on four amino acids from myelin basic protein (MBP) was designed to induce clinical disease in animals but was well tolerated with low/moderate efficacy on RRMS (32). Several orally administered drugs, including teriflunomide, provided moderate effects on RRMS (33, 34). Dimethyl fumarate and diroximel fumarate (35, 36), and fingolimod/FTY720 (37), the first approved oral drug for MS, had moderate beneficial effects on RRMS but with several side effects, including progressive multifocal leukoencephalopathy (PML). Modulators of sphingosine-1-phosphate receptor 1 (S1PR1) and S1PR5, and siponimod decreased oligodendrocyte and axonal loss (38). Ozanimod and SIPR1 and 5 agonist reduced plasma neurofilament light-chain concentrations (39), and the selective S1PR1 modulator ponesimod (40) and cladribine, a deoxyadenosine analog (41), provided moderate benefits to patients with MS. Notably, many intravenous infusion strategies were implemented for MS treatment. Mitoxantrone, a general immunosuppressant, was the first-line treatment with high efficacy for MS (42). The first humanized monoclonal antibody (mAb) used for MS treatment, natalizumab (43), is directed against anti-α4β1-integrins and blocks the entry of immune cells into the CNS. Although it is highly effective, it causes PML in John Cunningham virus-seropositive patients. The first humanized mAb, anti-CD52 antibody (campath-1/alemtuzumab), originally used for treating graft *vs*. host disease proved to be highly efficacious for MS treatment but associated with significant side effects (44, 45). Several B-cell-depleting anti-CD20 mAbs, such as rituximab, ocrelizumab, ofatumumab, and ublituximab, were highly efficacious for MS treatment but with PML occurrence in some cases (46–50). Most of these drugs were designed to regulate adaptive immune cells prominent during the early, but not the late, stage of MS (3, 27, 28). Some of these therapies reduce relapses but do not prevent the progression of MS and the accumulation of disabilities. The first-line treatments for MS, such as glatiramer acetate (32), dimethyl fumarate (35), and natalizumab (anti-IFN-β-1b) (43), affect T cells variously. Whereas IFN-β-1a/b reduced relapses without affecting Th17 cells (31), glatiramer acetate (32) and dimethyl fumarate suppress Th1 while upregulating Th17 cells (27). Alemtuzumab decreases central memory T cells (27). Fingolimod targets the SIPR and blocks T-cell transmigration into the CNS. This treatment results in cardiac complications, varicella–zoster, and herpes simplex virus reactivation, and exacerbation of MS (27, 28, 37). Natalizumab, a humanized monoclonal antibody, selectively targets the α4 subunit of the cell adhesion molecule, very late antigen 4, and prevents leukocyte adhesion and diapedesis at the blood–brain barrier, leading to PML in John Cunningham-virus seropositive patients (2, 3, 27, 28, 43). Systemic administration of anti-CD20 monoclonal B-cell-depleting antibody rituximab in patients with PPMS reduced gadolinium-enhancing lesions and relapses for 48 weeks (46). However, long-term therapy with ocrelizumab, a humanized depleting anti-CD20 mAb, provided modest protection against PPMS (47). Earlier and continuous treatment of patients with PPMS with ocrelizumab over 6.5 years provided sustained benefits on measures of disease progression (48). Since CNS B cells residing in meningeal ectopic lymphoid follicles are associated with subpial inflammation in patients with SPMS, inadequate penetration of the anti-CD20 antibody across the blood–brain barrier into the CNS could explain the lack of protection observed in some studies. Rituximab administered intrathecally also failed to provide clinical benefits in the phase 1b clinical trial on progressive MS (49). Other B-cell-depleting antibodies including ofatumumab (50) and ublituximab, a novel glycoengineered anti-CD20 mAb (51) that was administered SC unlike other mAbs, induced modest protection against MS.

Since 2018, several second-generation molecules with reduced gastrointestinal side effects have been approved for the treatment of MS by the FDA (52). Diroximel fumarate, the second-generation version of dimethyl fumarate, is lymphopenic and modifies monocytes. Oral formulations of S1PR modulators such as siponimod, ozanimod, and ponesimod target S1PR1 and SIPR5 have potentially better safety profiles. Ofatumumab, an anti-CD20 antibody administered subcutaneously, and glycoengineered anti-CD20 antibody, ublituximab, and oral compounds such as teriflunomide and cladribine were also approved for MS treatment (52).

Several other DMTs outnumbering those approved for MS treatment failed to meet the primary study endpoint and progress to a subsequent clinical trial because of commercial decisions. These include antibodies against the IL-12/23 p40 subunit (53), anti-CD25 (54, 55), CTLA-4-Ig (56), and anti-IL-17A (57). The mAbs targeting different subsets of B cells, tabalumab inhibited B-cell activation factor (BAFF), and atacicept induced depletion of mature B cells and suppressed antibody formation (58). However, they failed to deplete memory B cells and inhibit relapsing MS. Moreover, GNbAC1, a humanized mAb directed against an endogenous retroviral protein (59), and raltegravir (Isentress), the HIV integrase strand inhibitor (60), did not have an impact on MS disease activity. Interestingly, natalizumab failed to demonstrate a significant protective effect in patients with SPMS (61, 62). In addition, the anti-CD20 antibody, rituximab, shown to have superior protection in RRMS, has been abandoned due to the expiry of the patent (61, 62).

In addition to these non-specific drug therapies, several attempts were made to induce antigen-specific tolerance in encephalitogenic T cells, which would ensure stable and adequate protection against autoimmune diseases without off-target effects [reviewed in Refs. (63, 64)]. These include the administration of synthetic peptides corresponding to the T-cell epitopes mapped within myelin components such as MBP, MOG, proteolipid proteins (PLP), and altered ligand peptides. Moreover, T-cell receptor (TCR) vaccination constituting attenuated autologous antigen-specific T cells and autologous peripheral blood mononuclear cells chemically coupled with myelin peptides were also undertaken. None of these maneuvers induced T-cell tolerance as assessed by the ability of peripheral blood T cells to proliferate and produce IFN-γ in response to a challenge with the corresponding immunizing peptide *in vitro*. Significantly, they also did not improve the clinical outcome in patients with MS. Thus, effective methods of inducing antigen-specific tolerance in encephalitogenic T cells without causing adverse reactions remain an unmet need.

## Experimental models of MS

### Myelin antigen-induced experimental autoimmune encephalomyelitis

The MS-like disease, experimental autoimmune encephalomyelitis (EAE), has been successfully induced in monkeys, guinea pigs, rats, and mice, following immunization with the whole-brain and spinal cord extracts and peptides derived from myelin proteins, such as MBP, PLP, and MOG [reviewed in Ref. (65)]. The mouse is a popular choice for studying MS variations primarily due to the availability of genetically defined inbred strains and transgenic and gene knockout mice. SJL/J mice immunized with the PLP_139 − 151_ peptide or peptides derived from MBP exhibited relapsing-remitting EAE (RR-EAE) (66), and this model would allow the development of novel DMTs for RRMS. Immunization with rat MOG induced classic EAE in congenic C3H.SW (H-2^b^) mice, while causing atypical EAE characterized by ataxia, proprioception defects, and axial rotary clinical presentation in C3HeB/Fej (H-2^k^) mice (66, 67). Atypical EAE was also induced in IFN-γ knockout mice on the BALB/c background immunized with MBP-derived peptides (68). In one study, granulocytes were implicated in atypical EAE (66), while others found the participation of granulocytes in both classic EAE and atypical EAE (68). The brain seems primarily involved in atypical EAE, while the spinal cord is considered the primary target of classic EAE and RR-EAE (66, 68). Since the brain is primarily involved in MS (2–4), atypical EAE models may provide valuable tools for further understanding the mechanisms of brain lesions and their prevention.

MOG is a member of the immunoglobulin superfamily expressed exclusively in the CNS myelin. The MOG_35 − 55_ region proved to be an immunodominant epitope eliciting T- and B-cell responses and EAE in most strains of mice (65, 69–80). MOG_35 − 55_ was identified as an autoantigen in patients with MS (17). Immunization of C57BL/6 (H-2^b^) mice with the MOG_35 − 55_ peptide elicited EAE (78–80). However, MOG_35 − 55_ peptide immunization induced a robust and long-lasting progressive EAE (PEAE) in non-obese diabetic (NOD) (H-2^g7^) mice (70–77). Interestingly, pronounced remissions were observed in some (70–72), but not in other, studies (73–77), indicating variations in PEAE. Genetic drift and gene deletions could be attributed to the inconsistency in remissions in NOD mice bred in different geographical locations—Oceania, Europe, and the United States. The detection of T cells recognizing MOG_35 − 55_ peptide in patients with MS (17) provided an impetus to explore EAE specifically induced by this peptide autoantigen, although other myelin peptide antigens also elicited EAE in multiple strains of mice (65, 69) (Table 1). Moreover, NOD mice develop several autoimmune diseases, including type 1 diabetes (81) and other endocrine gland-related autoimmune conditions, such as thyroiditis, sialitis, and Sjögren's syndrome (82–84). Thus, NOD mice offer a unique opportunity to study the mechanisms of self-reactive T-cell-mediated neurodegeneration in an autoimmune environment. Significantly, PEAE induced in NOD mice lasts throughout the life of the mice with increasing disabilities (70–77), unlike the non-autoimmune-prone C57BL/6 mice (Table 1) (78–80). Biozzi ABH mice also develop PEAE when immunized with the whole spinal cord homogenate (85). Immunization of Lewis rats with gpMBP_68 − 84_ (86) and dark Agouti rats with MOG_1 − 125_ also induced classic EAE (87). Thus, EAE is a well-studied model system of MS and is amenable to investigating the efficacy of novel treatment options.

**Table 1:** Regulation of EAE by HDAC inhibitors.

**Model**	**Clinical manifestation**	**Drug**	**Drug administration**	**Clinical efficacy**	**Functional effect**	**Effects on gene expression**	**References**
C57BL/6	Acute, monophasic EAE	TSA, HDAC class I, IIa, and IV inhibitor-hydroxamate	Prophylactic—SC injection.	Reduced EAE.	Caspase inhibition.	Upregulation of genes encoding anti-oxidants, neuroprotection and neuronal differentiation.	(79)
C57BL/6	Acute, monophasic EAE	Vorinostat (SAHA)-HDAC class I and IIa inhibitor-hydroxamate	Prophylactic—intragastric, daily.	Reduced EAE.	Limits CNS inflammation and demyelination. Suppresses Th1, Th17 cells, and costimulatory molecules.	Not determined.	(80)
C57BL/6	Acute, monophasic EAE	Valproic acid, HDAC class I inhibitor	Prophylactic—day 3 or therapeutic-day 12 onward	Reduced EAE.	Suppression of spinal cord inflammation, demyelination, and T cells.	Reduction of caspase-3,−8, and−9 mRNA in T cells.	(81)
			—IP injection or oral administration.				
NOD	Primary, progressive EAE	TSA, HDAC class I, IIa, and IV inhibitor-hydroxamate	Prophylactic- days 0 to 45 or therapeutic- days 15 to 45-SC injection.	Diminished PEAE.	Reduced expansion and infiltration of granulocytes, Th1, Th1/17, and Th17 cells and their infiltration into the CNS.	Transcriptional repression of IL-17A, IL-27 p28, IL-27 Ebi3, iNos, and MIF in the peripheral lymphoid compartment.	(75–78)
					Diminished spinal cord inflammation, demyelination, and axonal loss.	Reduced transcription of IL-4, IL-17A, iNos, MIF, aryl hydrocarbon receptor, and Hdac11 but increased expression of DEC-1 mRNA in the CNS.	
					Induction of antigen-specific T cell tolerance.		
NOD	Primary, progressive EAE	Panobinostat, Givinostat (hydroxamate, pan-lysine inhibitor), and Entinostat	Therapeutic-day 20 onward-oral	No effect on PEAE or mortality.	Reduced T cell proliferation *in vitro*.	Reduced transcription of *Tbet* and *Rorgt* but not *Gata3* or *Foxp3* in lymphoid cells.	(78)
Lewis rat	Acute, monophasic	Valproic acid	Prophylactic and therapeutic-oral.	Reduced EAE	Th1/Th17-Th2 shift, attenuated infiltration of macrophages and lymphocytes in the spinal cord.	Suppressed mRNA levels of IFN-γ, TNF-α, IL-1β, MMP9, iNos, Tbet and increased IL-4 in the spinal cord.	(87)
Dark Agouti rat	Acute, monophasic	Valproic acid	Therapeutic-IP injection of multiple doses every day for many days.	A modest decrease in chronic EAE without affecting the peak response.	Reduced T cell proliferation and decreased Th17 cells.	Increased *Sox8* and *Mog* expression in the brain. Reduced demyelination in the spinal cord.	(88)

Mice were immunized with MOG_35 − 55_ peptide, Lewis rats with gpMBP_68 − 84_ peptide, and DA rats with MOG_1 − 125_ peptide.

### Other demyelinating disease models

Infection of mice with the neurotropic picornavirus Theiler's murine encephalomyelitis virus (TMEV) induces a disease similar to PPMS involving the brain, brainstem, and spinal cord (88). The TMEV infects macrophage/microglia, oligodendrocytes, and astrocytes during the chronic phase. Axonal damage in MS and EAE occurs secondary to inflammatory demyelination (outside-in model) (89). By contrast, TMEV infection induces demyelinating lesions that develop from the axon to the myelin (inside-out model) (90). Although TMEV infection cannot occur naturally in rodents or humans (91), it is a valuable model for studying the efficacy of drugs to prevent axonal degeneration independent of immune mechanisms. Feeding of C57BL/6 mice with the copper-chelating agent cuprizone induced demyelination, oligodendrocyte death, and profound activation of astrocytes and microglia (91). Removing cuprizone from the diet led to the regeneration of oligodendrocytes from the pool of oligodendrocyte progenitors and the formation of myelin sheaths, indicating the reversible nature of the disease. Interestingly, lysolecithin injection produced focal areas of demyelination in SJL/J mice, rats, and rabbits due to direct toxic effects on myelin sheath without affecting other cells and axons (91). These models help study the process of de- and remyelination independent of the involvement of immune mechanisms.

### EAE models for investigation of MS therapeutics

EAE models have traditionally been used to benchmark the efficacy of various disease-modifying therapies. However, several inconsistencies between mice and humans concerning the outcome of these attempts have been intensely debated (91–93). A few established MS therapies, including glatiramer acetate (copolymer 1), mitoxantrone, and natalizumab, were tested in animal models, which turned out to be potent non-specific suppressors and unsuitable for all patients with MS (27). Some DMTs were investigated in EAE models retrospectively after disappointing outcomes in human trials (91, 92). The failures of translational therapies for MS treatment could be due to differences in genetics, the extent of blood–brain barrier disruption, and individual variability in the responsiveness of patients to treatment. Emphasis has also been placed on discovering reliable biomarkers of MS and improving the design of CNS drug delivery (93). Most of the multifocal symptoms of classic MS have not been reproduced in rodent models. This limitation should be kept in mind when discussing the lack of efficacy of the DMTs for MS treatment since this disease is highly heterogenous and sometimes manifest with other comorbidities.

## Epigenetic approaches to control EAE

In EAE, adaptive immune T and B cells, the innate immune granulocytes, and the CNS-resident cells such as microglia, astrocytes, and oligodendrocytes collectively contribute to neurodegeneration. Gene expression is a highly regulated process, and aberrant expression of mRNA encoding cytokines and chemokines contributes to pathological manifestations. Although the genome-wide association studies have implicated genes encoding human leukocyte antigens in MS pathogenesis (94), environmental factors such as Epstein–Barr virus infection, smoking, and vitamin D deficiency may influence gene expression *via* epigenetic mechanisms (95). Epigenetics is the heritable changes in gene expression without altering the DNA sequence, which can provide a mechanism by which external factors, including drugs, produce various phenotypic variations with identical genotypes (96). Discordance in the rate of MS among monozygotic twins suggests that susceptible genes alone are not enough to manifest the neuronal disease, implying the participation of epigenetic mechanisms in disease manifestation (97). DNA methylation (98) and microRNAs (99) have been proposed to play a role in MS. However, direct evidence supporting the contention that modulation of these epigenetic mechanisms can result in neuroprotection is lacking.

Histone acetylation is the most well-characterized posttranslational mechanism of histone modifications, facilitating an open chromatin configuration and gene transcription (96) (Figure 1). The balance between acetylation by histone acetyltransferases and their regulation by histone deacetylases (HDACs) dictates the outcome of transcription of many protein-coding genes (96) and, interestingly, a non-coding microRNA (100). Trichostatin A (TSA), a hydroxamate member, was initially developed for cancer treatment (101) and is the most potent broad-spectrum HDAC inhibitor (102). TSA inhibits the transcription of class I, IIa, IIb, and IV HDACs (76). When C57BL/6 mice were immunized with MOG_35 − 55_ and treated with large doses of TSA s.c throughout the investigation, a modest reduction in the EAE score was noted (78) (Table 1). Similarly, daily oral administration of vorinostat, another hydroxamate that inhibits class I and IIa HDACs (102) throughout the period of investigation, also reduced the acute EAE in C57BL/6 mice (79). Interestingly, the class I HDAC inhibitor and the antiepileptic drug valproic acid when administered prophylactically or therapeutically reduced acute EAE in C57BL/6 mice (80). Notably, s.c administration of a lower dose of TSA prophylactically up to 45 days on alternate days provided irreversible and prolonged protection against PEAE in NOD mice (74). Consistent with these encouraging results of HDAC inhibitors to treat neurodegenerative diseases in mice, oral treatment of Lewis rats (86) or i.p administration of DA rats (87) with valproic acid reduced EAE induced by immunization with gpMBP_68 − 84_ and MOG_1 − 125_ peptides, respectively. In contrast to the success of reducing the clinical scores by TSA and valproic acid in C57BL/6 and NOD mice, oral administration of another hydroxamate panobinostat, givinostat, a pan-lysine inhibitor, or entinostat therapeutically from day 20 onward failed to afford protection against PEAE (77). These data indicate that not all HDAC inhibitors can serve as potent DMTs for ongoing neurodegeneration.

Neuroprotection provided by TSA, vorinostat (SAHA), and valproic acid corroborated with reduced CNS inflammation and demyelination in mice (74, 75, 79, 80). Significantly, inhibition of axonal degeneration during PEAE was also prominently mediated by TSA (74). Reduced T-cell proliferation and suppression of Th17 cells were noted in HDAC inhibitor-treated rodents (74, 79, 86, 87). Neuroprotection was also accompanied by decreased CD4^+^CD44^+^ cells, a characteristic of activated/memory cells (103), and reduced ability of T cells to produce IFN-γ, IL-17A, and GM-CSF in response to a challenge with MOG_35 − 55_ *in vitro* (74). Histone hyperacetylation rendered T cells unresponsive to the MOG_35 − 55_ antigen challenge while retaining their ability to respond to polyclonal stimulation (74), akin to anergy (104). By contrast, daily oral administration of HDAC inhibitors such as panobinostat, givinostat, and entinostat from the start of clinical signs (day 20) failed to protect NOD mice from PEAE or fatality, despite reduced T-cell proliferation *in vitro* and diminished transcription of *Tbet* and *Ror*γ*t* (77). However, the antiepileptic drug valproic acid (54) and the anti-cancer drug, TSA (74), administered therapeutically (after the disease onset, Table 1) provided robust neuroprotection and thus may be useful in a clinical setting.

## Regulation of the innate immune system in EAE by HDAC inhibitors

In MS, innate immune cells, such as infiltrating macrophages and dendritic cells, and CNS-resident microglia, have been implicated in the reactivation of T cells during the effector phase of neurodegeneration (2, 3). In NOD mice, PEAE development was associated with the expansion of *mature* (MHC class II^+^) CD11b^+^Ly-6G^+^ neutrophils and, to a lesser extent (MHC class II^+^) CD11b^+^Ly-6C^+^ mature monocytes in the peripheral lymphoid compartment before the onset of the peak clinical disease (75). Participation of neutrophils in monophasic EAE of C57BL/6 mice was indicated by increased neutrophils in the bone marrow, blood, and spleen during the early phase of the disease (105). Studies suggested a role for neutrophils in MS during the initial formation of lesions in the brain, but not during the advanced stages of the disease, probably owing to the short-lived nature of neutrophils (106). Treatment with TSA concurrently afforded neuroprotection and diminished the frequency of neutrophils in secondary lymphoid organs and their influx into the spinal cord (75), indicating a role for these cells in the PEAE model (Figure 1). Thus, in addition to myelin-specific T-cell tolerance induction, selective regulation of the innate immune system appears to be an integral part of the regulation of neurodegeneration by the HDAC inhibitor TSA.

## Implications of HDAC inhibitor-induced regulation of EAE to MS treatment

### Impact of immune regulation

Immune responses elicited by immunization with the whole spinal cord homogenate or various peptides derived from the CNS-associated MBP, PLP, and MOG have been extensively studied in mice and rats that develop monophasic EAE, PEAE, and atypical EAE (65–80, 86, 87). Various methodologies such as ELISA, Western blot, flow cytometry, and quantitative reverse transcriptase-mediated polymerase chain reaction (RTq-PCR) have provided significant insights into the underlying immune mechanisms of EAE. However, consensus on whether any given immune mediator can serve as a biomarker indicating the stage and severity of the chronic disease remains enigmatic. Most studies focused on immune mediators typically at the peak of the clinical disease after *in vitro* activation with T-cell ligands. A systematic and comprehensive analysis of *basal levels* of 41 genes frequently implicated in neurodegeneration and their regulation by TSA treatment was assessed using RTq-PCR in the CNS and secondary lymphoid organs longitudinally during the prolonged course of PEAE (27 weeks) without overt activation *in vitro* (76). These studies indicated that immunization of NOD mice with MOG_35 − 55_ increased the expression levels of mRNAs encoding IL-4 and IL-17A in the CNS during the chronic phase, days 21–54. The reduction in the level of IL-17A gene in TSA-treated mice is consistent with the proposed role of IL-17A in EAE (107). Prolonged expression of *Nos2* in the CNS (76) is in line with the association of iNOS-positive macrophages, astrocytes, and granulocytes in demyelinating pathology (108). Increased numbers of neutrophils in the spleen and spinal cord and their downregulation by the histone modifier treatment support this contention (75).

On the other hand, in the peripheral lymphoid tissues, genes encoding the heterodimeric chains of IL-27, IL-27p28, and IL-27EBi3, implicated in EAE (109), were overexpressed in PEAE mice, which were reduced by TSA treatment. Augmentation of the transcriptional repressors by histone acetylation could indirectly cause a reduction in gene expression. Notably, *in vitro* activation of peripheral lymphoid cells from TSA-treated mice exhibited compromised expression of both intracellular and secreted IL-17A and IFN-γ (74). Interestingly, TSA treatment reduced the infiltration of Th1 and Th17 cells from the periphery into the spinal cord (74) (Figure 1). This is similar to the suppressive effect of valproic acid on the influx of T cells into the spinal cord of EAE Lewis rats (86). These data demonstrate that the infiltration of T lymphocytes into the CNS is crucial for neurodegeneration, and their retardation by HDAC inhibitors facilitates neuroprotection.

Although migration inhibitory factor (MIF) has been proposed to be crucial for EAE (110), surprisingly, it was not transcriptionally upregulated in the CNS and lymphoid tissues of NOD mice manifesting PEAE (76). Yet, TSA treatment repressed the constitutive expression of *Mif* in protected mice. Surprisingly, several other genes implicated in EAE, including GM-CSF (111), prominent chemokine CCL2 (112), transcription factors T-bet (113), and RORγt (114), were neither overexpressed in the PEAE mice nor downregulated by TSA treatment (76). However, in EAE rats, valproic acid treatment suppressed the mRNA levels of IFN-γ, TNF-α, IL-1β, MMP9, iNos, and Tbet and increased IL-4 in the spinal cord (86). The transcription factor FoxP3 mRNA was neither upregulated in the PEAE model nor modulated by chromatin modifier treatment (76), similar to the lack of suppression of FoxP3 transcription in another study (77). TSA treatment also did not alter the numbers of FoxP3^+^ T regulatory cells in NOD mice (74, 76). Although the transcription factor FoxP3 is essential for the generation of T regulatory cells (115), it is contentious whether these cells are involved in the regulation of EAE (116, 117). Studies in mice indicated the upregulation of genes encoding anti-oxidants, neuroprotection, and neuronal differentiation by TSA treatment (78), while the expression of *Sox8* and *Mog* was upregulated in valproic acid-treated rat brains (87). Valproic acid administration also reduced the genes crucial for apoptosis, and caspase-3,−8, and−9 in T cells (78). Collectively, these data indicate that the HDAC inhibitors modulate the transcription of several genes crucially involved in neurodegeneration.

### The role of histone deacetylases in EAE and their modulation by TSA

Surprisingly, immunization of NOD mice with MOG_35 − 55_ upregulated the transcription of Hdac11 in the CNS, but none of the 11 Hdacs in the peripheral lymphoid cells (76). The wide-spectrum HDAC inhibitor, TSA, did not diminish the Hdac11 enzymatic activity *in vitro* (118), indicating the lack of correlation between Hdac expression and Hdac activity. Nevertheless, the data demonstrating the selective upregulation of *Hdac11* in the spinal cord of PEAE mice and its downregulation by TSA treatment have implications to the control of MS by histone modifiers. The use of high-resolution *in situ* hybridization and imaging revealed abundant expression of *Hdac11* in the hippocampus and Purkinje cells of rat brains, suggesting a role in locomotor activity and ataxic syndromes, respectively (119). However, it is unclear whether in PEAE mice, *Hdac11* expression is localized to these cells and downregulated by TSA treatment. Knockout of *Hdac11* reduced the infiltration of monocytes and myeloid DC into the CNS, expression of CCL2, clinical severity, and demyelination (120). Although both TSA treatment and *Hdac11* gene knockout resulted in amelioration of EAE, the protective effect of *Hdac11* deletion observed may be secondary to the absence of *Hdac11* in the CNS and unrelated to the impact on monocytes and CCL2 expression (120). Nevertheless, by extrapolation, repression of *Hdac11* could be beneficial in treating patients with MS with broad-spectrum HDAC inhibitors, such as TSA. Although *Hdacs* other than *Hdac11* was not regulated by the histone modifier either in the peripheral lymphoid tissues or in the CNS (74), HDAC3 mRNA was reportedly increased in the peripheral blood mononuclear cells of patients with RRMS (121). However, another study failed to validate this observation (122), indicating uncertainty of the role of *HDAC3* in MS. Interestingly, TSA treatment prevented the manifestation of type 1 diabetes in NOD mice associated with the transcriptional repression of *Hdac4, Hdac8*, and *Hdac9*, but not *Hdac11*, in the spleen (123). However, TSA administration did not influence the transcription of *Hdac* genes expressed in the target organ pancreas. These data suggest that the overexpression of specific Hdac is tissue- and disease-specific, which could be utilized to manipulate hard-to-treat diseases, including MS.

### Implications of HDAC inhibition to MS treatment

Targeting multiple HDAC isoforms might be necessary for specific indications and proof-of-concept studies. The involvement of specific HDACs crucial for various forms of MS has not yet been delineated. Studying the expression level of different HDAC genes in particular cell types in the secondary lymphoid organs and the CNS is essential for designing selective HDAC inhibitors for MS treatment. Based on the data obtained, it is possible to create more selective compounds that could prove safer by reducing off-target effects. In addition to the downregulation of many genes, the expression of the transcription factor Dec1 (Bhlhe40) was upregulated in the CNS of TSA-treated mice (76). Thus, HDAC inhibitors such as TSA with broad specificity might provide benefits against complex neurodegenerative diseases by concurrently repressing and increasing the transcription of multiple genes. The wide range of the action of the broad-spectrum HDAC inhibitor is likely to provide protection against complex neurodegenerative diseases like MS. Consistently, therapeutic intervention with HDAC inhibitors has been proposed to enhance synaptic plasticity, learning, and memory in Alzheimer's disease, Huntington's disease, and Parkinson's disease (124). Lysine acetylation of non-histones constitutes a significant portion of the acetylome in mammalian cells and is involved in several cellular functions, including gene transcription (125). However, it is unclear whether HDAC inhibitors can also acetylate non-histones and alter gene transcription in conjunction with gene regulation mediated by acetylation of histone tails. Nevertheless, changes in gene expression due to inhibition of HDACs by small-molecule inhibitors could have substantial impact on regulating disease pathogenesis.

Recent work has unraveled the inheritance of non-DNA sequence-based epigenetic information, epimutations, across several generations in yeast to humans (126). The signals that underpin these epimutations, including DNA methylation, histone modification, and non-coding RNAs, and the underlying mechanisms are beginning to be understood (127). Treatment of the nematode *Auanema freiburgensis* with class I HDAC inhibitors butyrate and valproic acid, and the broad-spectrum HDAC inhibitor TSA increased the acetylation of histones 3 and 4 (128). Notably, they also exerted transgenerational effects on the offspring by producing increased numbers of hermaphrodites, suggesting that histone acetylation represents the histone code. The HDAC inhibitors have successfully ameliorated several diseases, including type 1 diabetes (123, 129–132), EAE (74–76), asthma (133), lupus (134, 135), and colitis (136), in animal models, indicating their usefulness to treat a variety of diseases. Accumulating data indicate that histone modifier-mediated hyperacetylation in lymphoid cells and the target tissues is associated with the amelioration of type 1 diabetes (129) and PEAE (74), and selective regulation of genes. It remains to be seen whether the changes in gene expression observed following treatment with HDAC inhibitors have transgenerational consequences.

## Conclusion

This review discusses the effects of HDAC inhibitors on EAE regulation (Table 1) and, by extrapolation, their utility in treating MS. Neuroprotection in mice was accompanied by the repression of mostly non-overlapping sets of genes induced by immunization with myelin antigens and a few constitutively expressed genes in the peripheral lymphoid system and the CNS. Notably, TSA administration contrived the expansion of granulocytes and induced T-cell tolerance in the periphery while reducing the influx of immune cells into the CNS (Figure 1). Lessons learned from the EAE models require validation, which may provide impetus to investigate the efficacy of histone modifiers for treating MS variants efficiently. Since HDAC inhibitors such as valproic acid and hydroxamates are currently used in patients for ailments unrelated to MS and are well tolerated, these small-molecule inhibitors may be used for treating MS.

## Author contributions

SJ conceived and executed the project and wrote the first draft of the manuscript. AJ conducted most of the experiments reported in this article and edited the manuscript. All authors contributed to the article and approved the submitted version.

## Funding

The work described in this article was supported by the University of Illinois at Chicago.

## Conflict of interest

The authors declare that the research was conducted in the absence of any commercial or financial relationships that could be construed as a potential conflict of interest.

## Publisher's note

All claims expressed in this article are solely those of the authors and do not necessarily represent those of their affiliated organizations, or those of the publisher, the editors and the reviewers. Any product that may be evaluated in this article, or claim that may be made by its manufacturer, is not guaranteed or endorsed by the publisher.
